# Cloning and Expression of β-Defensin from Soiny Mullet (*Liza haematocheila*), with Insights of its Antibacterial Mechanism

**DOI:** 10.1371/journal.pone.0157544

**Published:** 2016-06-20

**Authors:** Zhitao Qi, Wei Xu, Fancui Meng, Qihuan Zhang, Chenglung Chen, Rong Shao

**Affiliations:** 1 Key Laboratory of Biochemistry and Biotechnology of Marine Wetland of Jiangsu Province, Yancheng Institute of Technology, Yancheng, 224051, Jiangsu, China; 2 Key Laboratory of Aquaculture and Ecology of Coastal Pool in Jiangsu Province, Yancheng Institute of Technology, Yancheng, 224051, Jiangsu, China; 3 Tianjin Institute of Pharmaceutical Research, Tianjin, 300193, China; 4 Department of Chemistry, National Sun Yat-Sen University, Kaohsiung, 80424, Taiwan, ROC; Chinese Academy of Sciences, CHINA

## Abstract

Beta-defensins are important part of innate immunity of fish, which are the first defense line against invading pathogens. In this study, the β-defensin (Lhβ-defensin) gene was cloned from spleen tissue of soiny mullet (*Liza haematocheila*). Lhβ-defensin cDNA was 747 bp in length, encoding 63 amino acids. Sequence alignment revealed that Lhβ-defensin contained six conserved cysteine residues and shared 97.5% sequence identities with grouper (*Epinephelus coioides*) β-defensin. Realtime PCR revealed that Lhβ-defensin was highest expressed in the immune related organs, such as spleen, kidney and gut of healthy fish. Following *Streptococcus dysgalactiae* infection, Lhβ-defensin was up-regulated in immune related organs, e.g. 17.6-fold in spleen and 10.87-fold in gut at 24 h post infection (hpi). Lhβ-defensin possessed a monomeric structure of a three-stranded anti-parallel β-sheet and an α-helix stabilized by three disulfide bonds formed by Cys30-Cys58, Cys36-Cys52, and Cys40-Cys59. In addition to the experimental work, computer simulation was also carried out to determine the possible conformation of β-defensin and its interaction with palmitoyloleoylphosphatidylglycerol (POPG), a model of bacteria membrane. The Lhβ-defensin was found to form dimeric structure stabilized by the van der Waals contacts of Leu35 and Cys37 in two anti-parallel β1-strands and the cation-π interaction between Tyr32 and Arg54 respectively in the two β1-strands. The most important interactions between β-defensin and membrane are the electrostatic interactions between Arg residues in β-defensin and head group of POPG bilayer as well as hydrogen bond interactions between them. Our results were useful for further understanding the potential mechanism of antimicrobial property of fish β-defensins.

## Introduction

Antimicrobial peptides (AMPs) are important components of the innate immunity acting as the first defense line against invading pathogens. The defensins are cysteine-rich cationic antimicrobial peptides which are divided into three distinct groups, α-, β- and θ-defensins, based on the different disulfide bond [1,2]. α-defensins were isolated from mammalian neutrophils and macrophages which are stabilized by a mode of disulfide bonds of C1-C6, C2-C4 and C3-C5. θ-defensins are the macrocyclic product of a head-to-tail of two truncated α-defensins which are only found in leukocytes and bone marrows of Old World monkeys [2, 3]. β-defensin are the largest group of defensins family and are the most characteristic defensins. β-defensins are comprised of 35 to 50 amino acids with a core of three anti-parallel β-strands stabilized by a mode of disulfide bond of C1-C5, C2-C4 and C3-C6 [4].

β-defensins had been characterized in a number of fish species, such as zebrafish (*Danio rerio*), fugu (*Takifugu rubripes*), tetraodon (*Tetraodon nigroviridis*) [5], blunt snout bream (*Megalobrama amblycephala*) [6] and Nile tilapia (*Orechromis niloticus*) [7]. Fish β-defensins exhibited similar functions as mammalian β-defensins, including antimicrobial activity against bacterial [8, 9] and viruses [10], chemotactic attractants of immune cells [11] and modulator of immune response [12, 13].

Many studies had been carried out to elucidate the mechanism of action of various mammalian AMPs. The positively charged surface of defensins played a key role in their actions, which enabled them to initially attach to the bacterial membrane, destabilize and disrupt the cell membrane and format pores on membrane resulting in the leakage of the cell contents [14–16]. However, the diversity of sequences and structures of AMPs revealed that these molecules might act in diverse ways [16]. And so far, there were less studies on the mechanism of the fish AMPs. Thus, the present study described the successful cloning and characterization of β-defensin from soiny mullet (*Liza haematocheila*), an economically important aquaculture mugilid species in China and other Asian countries [17]. Since it is very difficult to determine experimentally the structure of Lhβ-defensin, computational method was adopted to probe into monomeric and dimeric structure of Lhβ-defensin by using protein docking and followed by molecular dynamics (MD) stimulation. The interaction of Lhβ-defensin dimer and bacterial membrane (POPG lipid membrane) was also investigated base on the MD simulation.

## Materials and Methods

This study was carried out in strict accordance with the approval of the Academic committee of Yancheng Institute of Technology (No. 2015016). The fish were euthanized by using ethyl-3 aminobenzoate methanesulfonate (MS-222, Sigma) according to the Measures for the Administration of Experimental Animals in Jiangsu province (No. 2008[45]). This study did not involve endangered or protected species. The *Streptococcus dysgalactiae*, a kind of gram positive bacterial, was kindly provided Professor Li Aihua from the State Key Laboratory of Freshwater Ecology and Biotechnology, Institute of Hydrobiology, the Chinese Academy of Sciences.

### Fish

Healthy soiny mullet with an average weight of 5.0 ± 0.5 g were purchased from Dafuyuan mill of fishery (Sheyang town, Yancheng city, China) and transported to our laboratory by an oxygen-supplying car. The fish were reared in 120 L plastic aquaria supplied with oxygen and fed with commercial diet twice daily. The acclimation lasted for 2 weeks prior to experiments.

### Molecular cloning of Lhβ-defensin gene

Total RNA was extracted from spleen tissue using TRIzol reagent according to manufacturer’s instructions. After the quality verification of RNA by electrophoresis and spectrophotometry, RNA was reverse transcripted into first-strand cDNA using First Strand cDNA Synthesis Kit (Fermentas, USA) according to manufacturer’s constructions.

Degenerate primers (LhBD-dF1 and LhBD-dR1) were designed based on the conserved regions of teleost β-defensin genes (Table 1). PCR was carried out using degenerate primers with spleen cDNA as template to obtain the partial sequence of Lhβ-defensin. The PCR products were ligated into pMD18-T vector (TaKaRa, Japan) and then transformed into *E*. *coli* DH5α competent cells. The selected positive clones were used for subsequent sequencing (Sangon Biotechnology Company, Shanghai, China). After the sequence was suggested to be the partial sequence of β-defensin gene by BLASTx analysis, specific primers were designed to obtain the full length cDNA sequence of Lhβ-defensin gene by 5' and 3' rapid amplication of cDNA ends (RACE) PCR method. The primers used in this study were listed in Table 1.

**Table 1:** Primers used for gene cloning and expression analysis.

*Primer*	*Sequence (5' to 3')*	*Application*
LhBD-dF1	ATGAAGGGACTGAGCTTGGTTC	RT-PCR
LhBD-dR1	GTGATG(CA)CCAACG(AG)TGTACTCCTG	RT-PCR
LhBD-5out	ACCCACAGGTCCAATACTGCAT	5'RACE PCR
LhBD-5in	AGAACCGTCTGCAGAGTCCTCA	5'RACE PCR
LhBD-3out	ATGCAGTATTGGACCTGTGGGT	3'RACE PCR
LhBD-3in	AGAGGACTCTGCAGACGGTT	3'RACE PCR
LhBD-F	CAGTATTGGACCTGTGGG	Realtime PCR
LhBD-R	CTAAGACCGCACCGCACA	Realtime PCR
Lhactin-F	CAGCCATACTGTGCCCATCT	Realtime PCR
Lhactin-R	TCCTTGATGTCACGCACGAT	Realtime PCR

### Sequence analysis

The amino acid (aa) sequence was deduced by Translate software in the ExPasy website (http://www.expasy.org). The alignment of amino acid sequences was carried out using ClustalO software (http://www.ebi.ac.uk/tools/msa/clustalo/), and decorated with BoxShade software (http://www.ch.embnet.org/software/BOX_form.html). The amino acids sequence identities were analyzed using MegAlign software in the DNAStar package. The phylogenetic tree was constructed by MEGA 4.1 software using neighbor-joining algorithm in which the bootstrap was set as 10,000 replicates to measure the confidence of branch topology [18].

### Tissue distribution of Lhβ-defensin transcripts

Four tissues including liver, spleen, kidney and gut were sampled from six healthy fish, respectively. RNA extraction with TRIzol reagent, cDNA synthesis and realtime quantitative PCR were carried out as described previously [19]. The expression level of Lhβ-defensin was normalized to that of β-actin. Primers used for real-time quantitative PCR were listed in Table 1.

### Expression of Lhβ-defensin following *Streptococcus dysgalactiae* infection

The bacterial infection experiments were carried out on the basis of our previous studies [17, 19]. Briefly, twenty fish were randomly divided into two groups including control group in which fish were infected intraperitoneally (i.p) with 100 μL PBS, and bacterial infection group in which fish were injected with 100 μL live *S*. *dysgalactiae*, (2×10^6^ CFU). The fish were monitored every two hours and no dead fish was observed during the whole experiment. The tissues including liver, spleen, kidney and gut from nine fish per group were sampled respectively at 24 h post infection (hpi). Real-time PCR was done as described previously and expressed as fold change relative to the time-matched controls [19].

### Homology modeling and MD analysis of Lhβ-defensin monomer and dimer

The monomeric structure of Lhβ-defensin was constructed by homology modeling method using Prime module of Schrödinger software [20] with the available human β-defensin-1(HBD-1) structure (PDB ID: 1IJV) as template [21]. Afterward, the dimeric structures were generated using SymmDock server [22, 23]. The generated 100 structures were clustered according to RMSD (root mean square deviation) values and four typical dimeric structures were obtained. Then, molecular dynamics simulations of the four typical dimers were performed using GROMACS 5.1 software [24] and AMBER99SB force field [25]. Briefly, the initial dimeric structure was placed in a box with an edge of 1.2 nm and solvated with TIP3P (three-point transferable intermolecular potential) water molecules. Chloride ions were added to neutralize the system. After energy minimization, 100 ps NVT and 100 ps NPT MD simulations were performed with position restrictions on peptides. Finally, MD simulation was carried out for 20 ns with a time step of 1 fs and trajectory was saved every 20 ps for analysis. The V-rescale method was used for temperature coupling with the reference temperature 300 K. The pressure was controlled isotropically using Parrinello-Rahman coupling method. Particle mesh Ewald (PME) method was used to consider the long-range electrostatic interaction. The cut-off distances for electrostatic and van der Waals interactions were both set to be 1.4 nm.

### Interaction between Lhβ-defensin dimer and POPG bilayer

The palmitoyloleoylphosphatidylglycerol (POPG) bilayer was constructed to assemble the bacterial membrane. Both the coordinate and the topology parameters for POPG bilayer were obtained from lipidbook (http://lipidbook.bioch.ox.ac.uk) [26]. This lipid bilayer includes 128 POPG molecules and has been tested in previous reported calculations [27]. The calculated order parameters, area per lipid and thickness of this bilayer with the AMBER force field were in excellent agreement with experiments [28, 29]. MD simulation was carried out to investigate the interaction between Lhβ-defensin and the membrane. The dimeric structure of Lhβ-defensin was placed in a random orientation about 5 nm away from the POPG bilayer center. Then, the system was solvated with roughly 10,000 water molecules and counter ions were added to neutralize the system. MD simulation was performed on this system for 100 ns. The reference temperature was set at 323 K, which is higher than the phase transition temperature of POPG bilayer. All the calculations were carried out using GROMACS package 5.1 [24] with AMBER99SB force field [25].

## Results and Discussion

### Sequence features of Lhβ-defensin

The cloned Lhβ-defensin cDNA was 747 bp in length (GenBank accession no. KJ872680), including 5'-untranslated region (UTR) of 215 bp, open reading frame (ORF) of 192 bp, and 3'-UTR of 340 bp. There was one polyadenylation signal (AATAAA) upstream of the poly (A) tail (S1 Fig).

**Fig 1:**
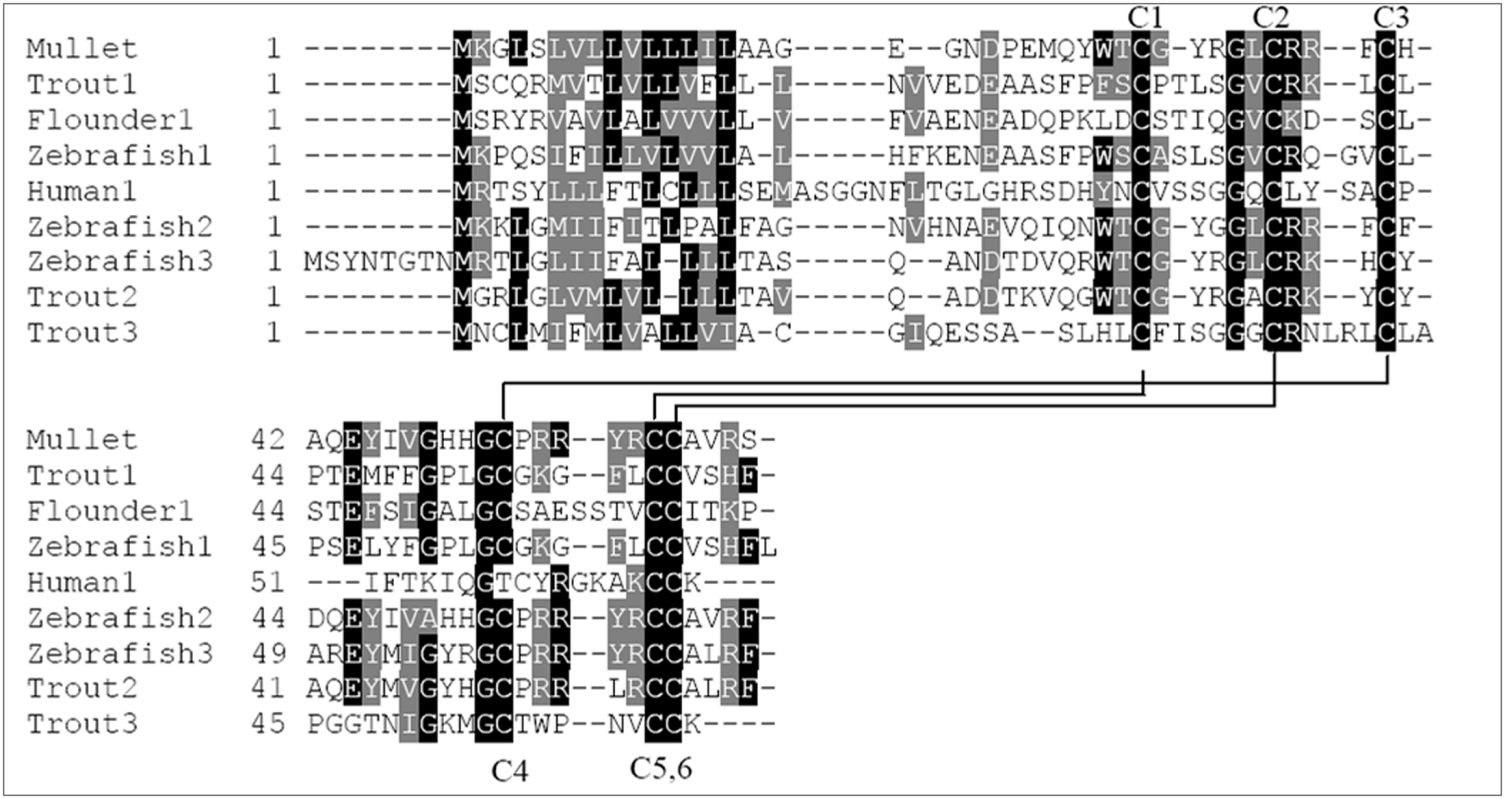
Multiple alignment of vertebrates β-defensins. C1-C6 indicated the six conserved cysteine residues. Black shade indicated identical amino acids and gray shade indicated similar amino acids.

The fish β-defensins ranged from 62 to 68 amino acids in length and contained a signal peptide of 18–26 amino acids [30]. In the present study, the ORF of Lhβ-defensin cDNA encoded 63 amino acids, which shared similar length with known fish β-defensin pro-peptide. A signal peptide of 20 amino acids was also predicted from Lhβ-defensin using SignalP software. Thus, the mature peptide of Lhβ-defensin contains 43 amino acids. The molecular weight of mature peptide was 5.18 kD and the isoelectric point (pI) 8.92, indicating that the net cationic feature of Lhβ-defensin. Further sequence alignment revealed that Lhβ-defensin shared 22.5–97.5% sequence identities with teleost β-defensins, among which highest sequence identities was with grouper (*Epinephelus coioides*) β-defensin (97.5%) and lowest with defensin-4 of Japanese flounder (*Paralichthys olivaceu*s) (22.5%). Six conserved cysteine residues were found to exist in Lhβ-defensin (Fig 1), which were well conserved in insects, fish, chicken and human β-defensins, and were crucial for structural stability and functions of β-defensin [31].

### Phylogenetic tree analysis

**Fig 2:**
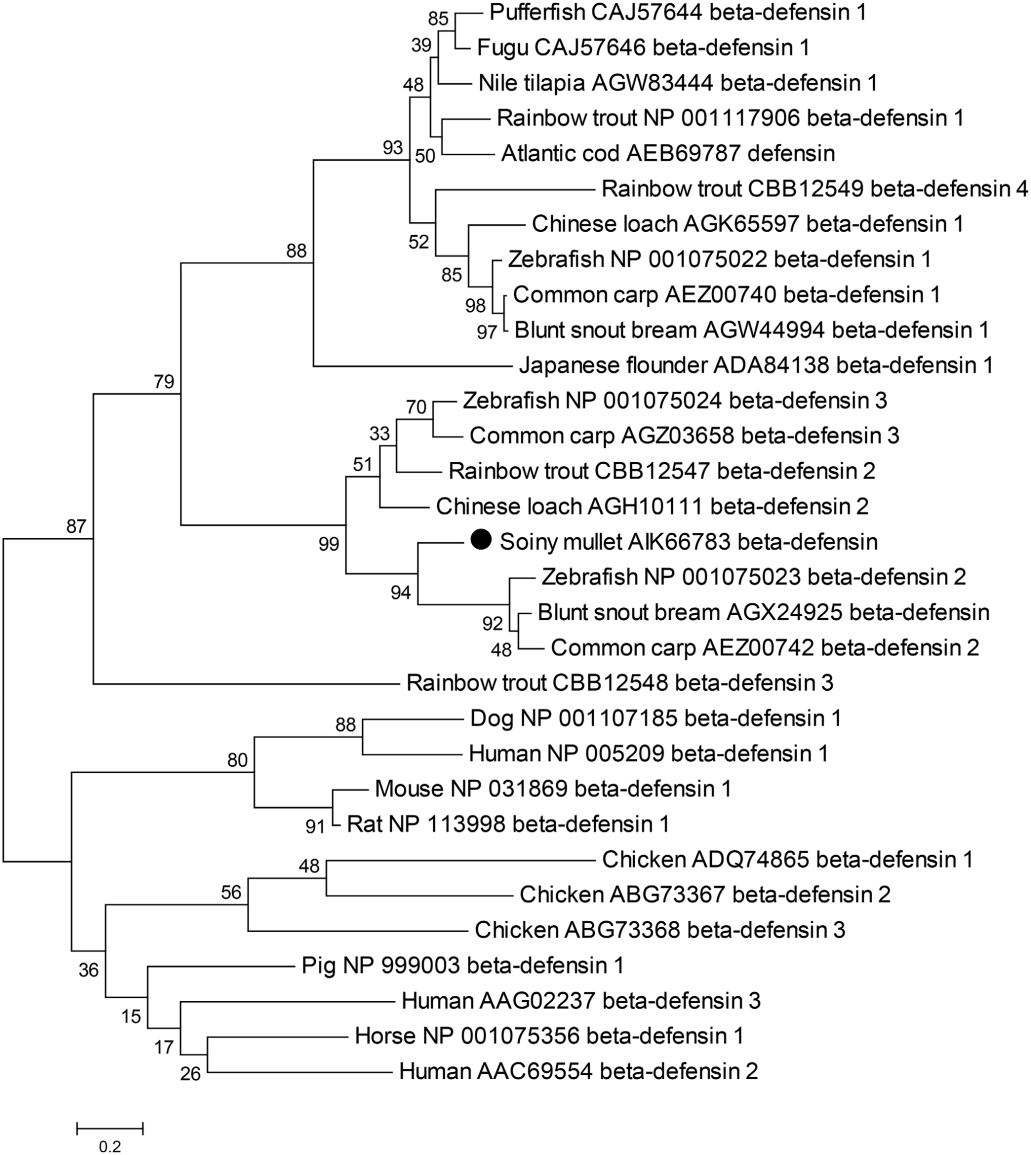
Phylogenetic tree of veterbrates β-defensins. The tree was constructed by MEGA 4.1 software using neighbor-joining method in which the bootstrap was set as 10,000 replicates to measure the confidence of branch topology. The Genbank accession number of sequences used for the tree was listed behind the common name of species. The newly obtained Lhβ-defensin was marked by round black cycle in the tree.

A neighbor-joining phylogenetic tree was constructed with the reported β-defensin amino acids sequences of veterbrates using MEGA4.1 software. Results revealed that the phylogenetic tree was divided into two main clusters. One cluster contained mammalian and birds β-defensins and another one contained the fish β-defensins. The cluster of fish β-defensins was further divided into two sub-clusters, one sub-cluster including all members of fish β-defensin1, and another sub-cluster containing fish β-defensin 2 and 3. The Lhβ-defensin was placed well in the sub-cluster of fish β-defensin 2 and 3. Similar results were also observed in the cluster of mammalian β-defensins (Fig 2). β-defensins in fish had expanded and diverged into two separated types. These two separated types of β-defensins may have diverged before the appearance of fish species and arisen from a common ancestor by gene or genome duplication events [5]. Multiple and divergent β-defensins may help animals to better defense against the diverse microbial pathogens in their habitats [32].

### Expression pattern of β-defensin in tissues from healthy and *S*. *dysgalactiae* infected mullets

**Fig 3:**
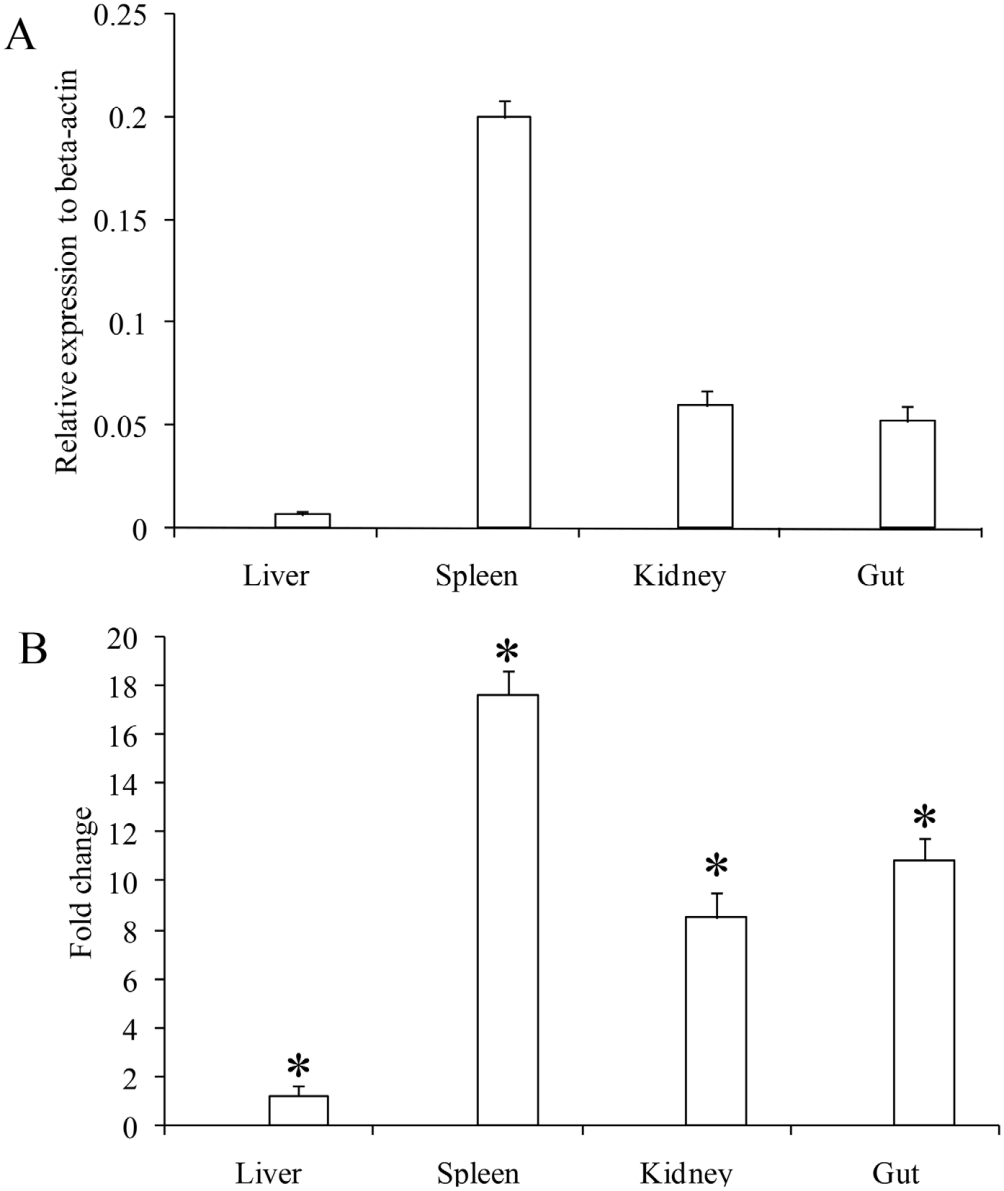
Tissues expression of Lhβ-defensin in tissues of healthy and *S*. *dysgalactiae* infected fish. (A) Expression of Lhβ-defensin in healthy fish was measured by realtime PCR and normalized to β-actin. (B) Expression of Lhβ-defensin in fish infected with *S*. *dysgalactiae*. Fish in the bacterial infected group were infected intraperitoneally with 2×10^6^ CFU live *S*. *dysgalactiae* and the control group was injected with the same amount of PBS solution. At 24 hpi, the tissues were selected for the gene expression analysis by Realtime PCR method. Asterisks indicate that the expression of Lhβ-defensin in the infected group was significantly up-regulated compared with that of control group (*P<0.05).

Mammalian β-defensins were mainly expressed in the epithelia of many organs while fish β-defensins were mainly expressed in the immune and mucosal tissues. However, expression patterns of fish β-defensins were different in various fish species, e.g mandarin fish β-defensin was highly expressed in the spleen, intestine, gill and head-kidney [9], and Wuchang bream β-defensin1 was highly expressed in skin, blood and immune related organs [6], whist Nile tilapia β-defensin was highly in skin, spleen, kidney and muscle [7]. In the present study, Lhβ-defensin was highest expressed in spleen, followed by the kidney and gut, and lowest in liver (Fig 3A). Thus, it was suggested that fish β-defensins may be species-specific and play a broad role in different fish tissues.

The expression of fish β-defensins was modulated by microbial pathogens infections. Following *Aeromonas sobria* infection, the expression of β-defensin was up-regulated in the skin of Wuchang bream [6]. Rainbow trout β-defensins were also up-regulated in the intestine and gill at 48 h post of *Yersinia ruckeri* infection [33]. In addition, Nile tilapia β-defensin exerted different up-regulated pattern at different time points post *S*.*agalactiae* infection, e.g. 3.3-fold in skin at 6 hpi, 57.1-fold in muscle at 24 hpi and 1.6-fold in gill at 72 hpi [7]. Similar to previous reports, Lhβ-defensin was also up-regulated following *S*. *dysgalactiae* at 24 hpi, e.g. 17.6-fold in spleen, 10.87-fold in gut and 1.23-fold in liver (Fig 3B). The up-regulated β-defensin may take part in the immune response against invading pathogens.

### Monomeric and dimeric structure of Lhβ-defensin

**Fig 4:**
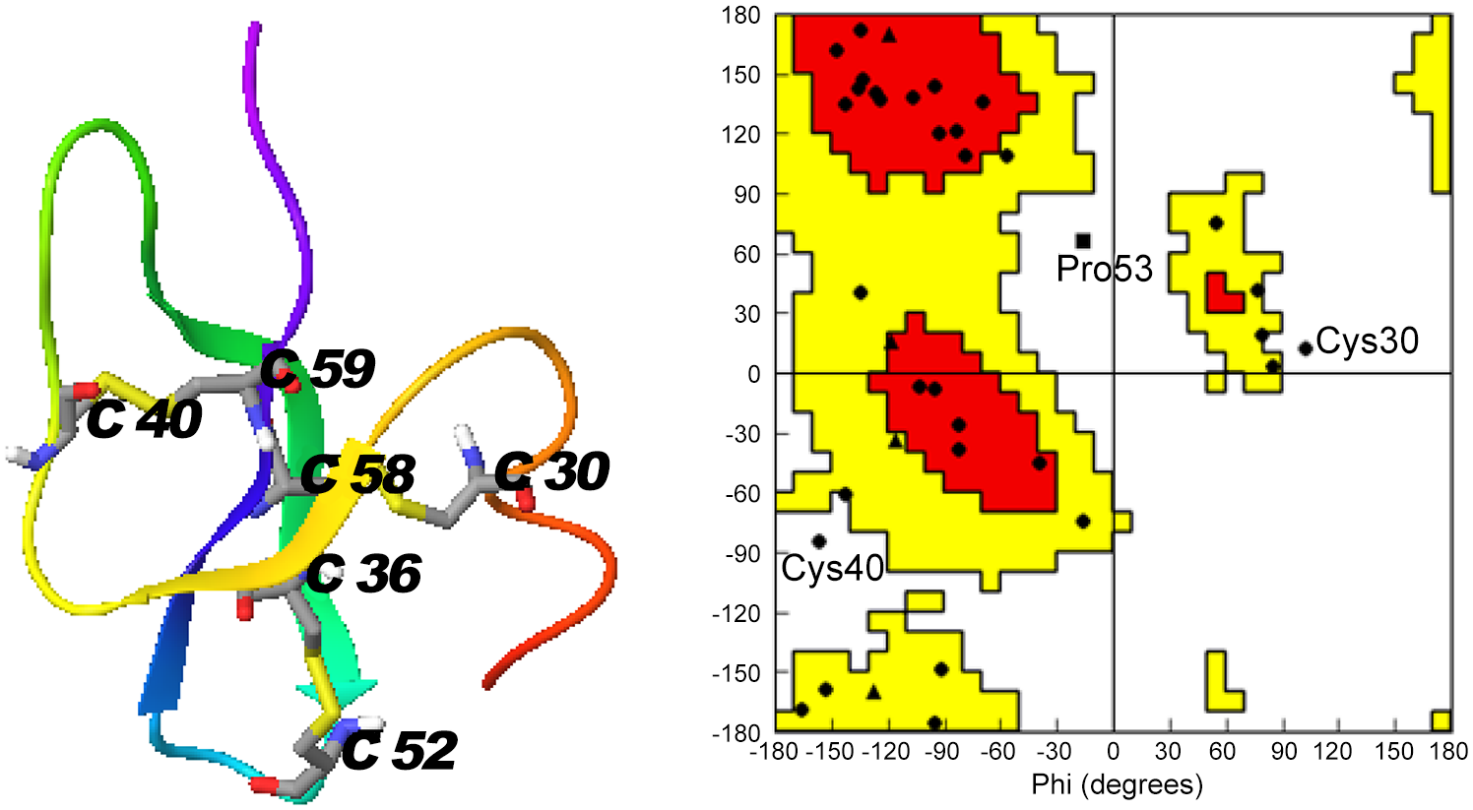
Predicted monomeric structure (A) and Ramachandran plot (B) of Lhβ-defensin.

The monomer of Lhβ-defensin shared a similar structure with human β-defensin2 [34], possessing a three-stranded anti-parallel β-sheet and an α-helix stabilized by three disulfide bonds formed by Cys30-Cys58, Cys36-Cys52, and Cys40-Cys59 (Fig 4A). The mode of disulfide bonds in this monomer was perfect matched the mode of disulfide bonds of β-defensin family members, which confirmed the gene we cloned was exactly the homologue of β-defensin. The results of Ramachandran angle analysis of this monomer showed that most of residues appeared in the energetic favored and allowed regions, and only three residues occurred in the disallowed region (Fig 4B). This indicated that the constructed structure was reliable.

**Fig 5:**
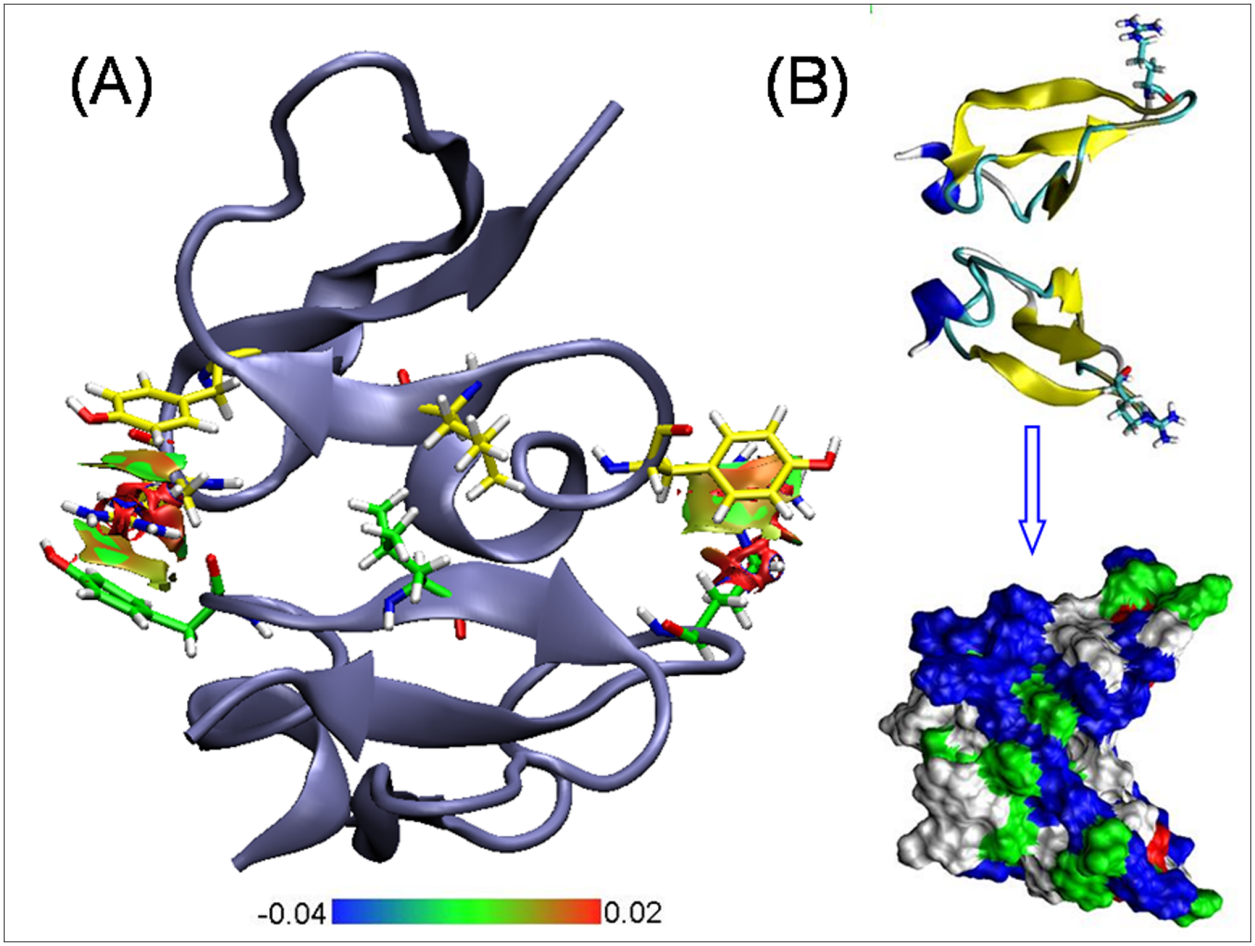
Reduced density gradient isosurface and residue type of Lhβ-defensin dimer. (A) Reduced density gradient isosurface map between Arg54 and Tyr32 in dimeric structure of Lhβ-defensin created by Multiwfn software [36]. The blue, red and green (or earth green) colors indicate the strong attractive, strong repulsive and van der Waals interaction, respectively. (B) Surface of dimer colored by residue type. Blue are basic residues, red are acidic residues, green are polar residues, and white are non-polar residues.

Further, the dimeric structures of Lhβ-defensin were constructed using SymmDock method. Four typical dimers of Lhβ-defensin were obtained after clustering by RMSD analysis. Dimers A and B were formed by parallel and anti-parallel β1–β1 strands interactions while dimers C and D were created through parallel and anti-parallel β2–β2 strands interactions, respectively (S2 Fig). Then, the stability of each dimeric structure was analyzed by equilibrium MD simulation and solvent accessible surface areas analysis using GROMACS 5.0 software. Dimer C had the largest RMSD value among the four dimers and its RMSD fluctuated widely compared with other dimers, revealing that the structure of dimer C deviated seriously from its initial structure and did not reach equilibrated state after 20 ns MD simulation. The overall RMSD of dimer B was about 0.3 nm with moderate fluctuation after 10 ns simulation, suggesting that it was well equilibrated during MD analysis. The RMSD profiles of dimer A and D were similar and their RMSD values ranged in the middle of that of dimers B and C (S3 Fig). Further solvent accessible surface areas revealed that dimer B had the largest buried surface and oscillated at 12 nm^2^, while the buried surface areas of the other three dimers fluctuated at 10 nm^2^. Significant fluctuation was observed for Dimer C, revealed large structure alteration occurred during the simulation (S4 Fig). Thus, it was reasonable to choose the dimer B for further analysis. The interface of dimer B was formed through van der Waals contacts of Leu35 and Cys37 in two anti-parallel β1-strands. The overlap of β1 strands was stabilized by the cation-π interactions between Tyr32 and Arg54 respectively in the two β1-strands (Fig 5A and 5B). Tyr56 also participated to create cation-π interactions with Arg54. Cation-π interaction is a non-covalent molecular interaction between the electron-rich π system and a positively charged species. The hydrophobic residues create a large surface of dimer B, including residues Tyr27, Cys30, Gly31 and Tyr 32 in α-helix, Gly34, Leu35 and Cys36 in β1 strand, and Pro53 in the loop between β2 and β3 strands (Fig 5B). The buried hydrophobic surface increased the positively charged surface during formation of dimer B, which play a crucial role in stabilizing protein structure and may enhance the interaction of β-defensin with negatively charged membrane [35].

### Insights of antibacterial mechanism of Lhβ-defensin

**Fig 6:**
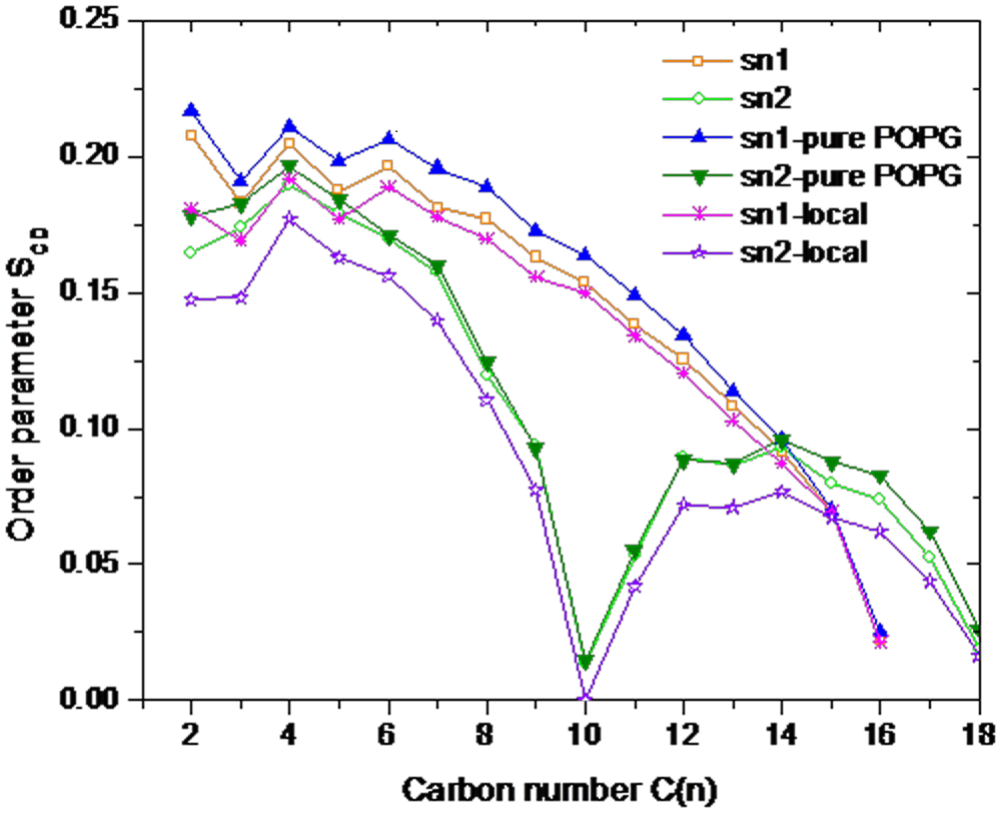
Order parameters (S_CD_) of saturated (sn-1) and unsaturated (sn-2) hydrocarbon chains in POPG computed using the last 20 ns trajectories of stimulation. Sn-1 and sn-2 referred to S_CD_ calculated by POPG in the presence of Lhβ-defensin dimer, sn1-local and sn2-local to S_CD_ calculated when PGPG was around 10 Å of defensin dimer, while sn1-pure POPG and sn2-pure POPG to S_CD_ calculated by POPG in the absence of Lhβ-defensin dimer.

**Fig 7:**
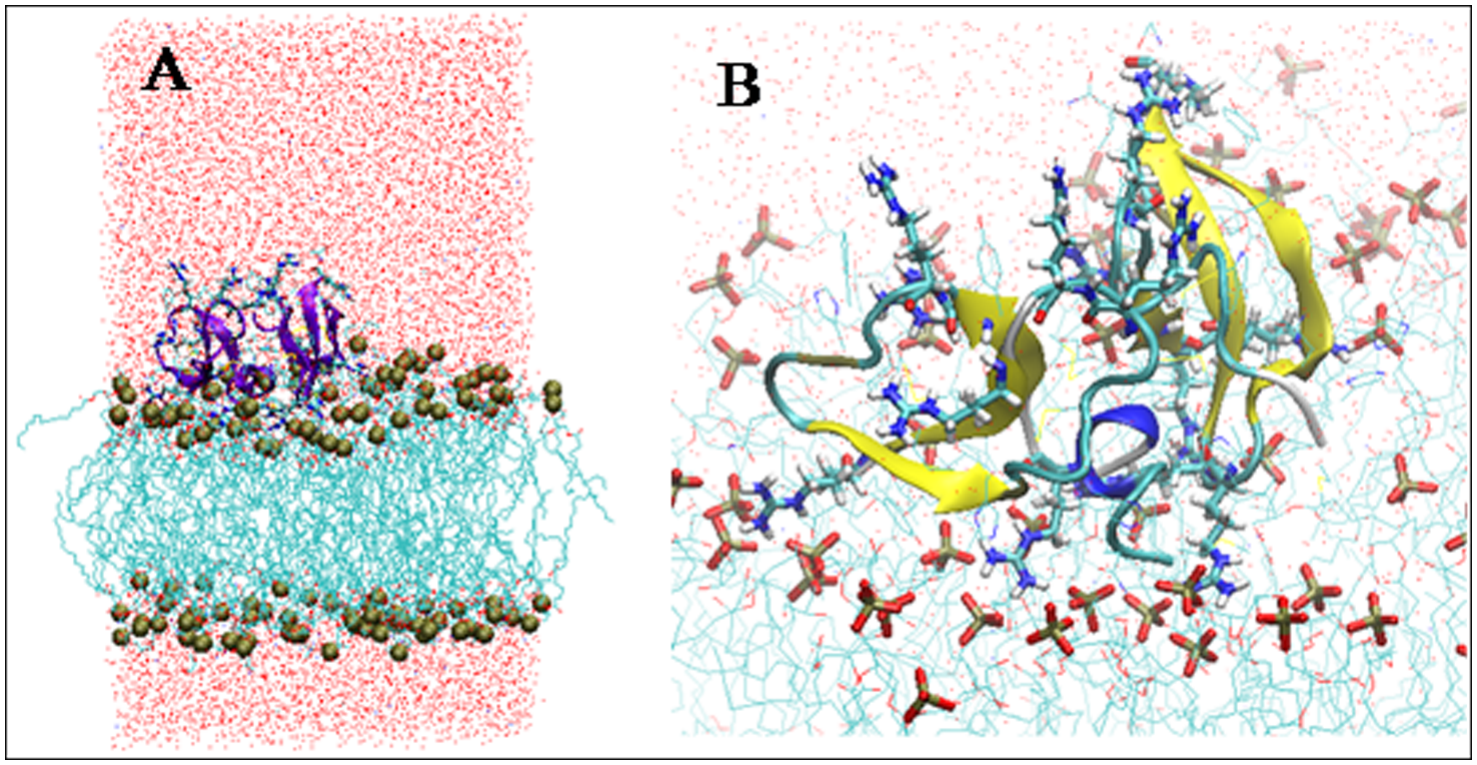
Snapshot showing the water defects and water translocations across the membrane (A); Interactions between arginines of Lhβ-defensin and POPG bilayer (B).

To reveal the potential antibacterial mechanism of Lhβ-defensin, the structure order of acyl chains of POPG membrane, one of model of bacterial membrane [37], with or without Lhβ-defensin dimer were analyzed [38]. There was a decreasing in the order parameters of atom 10 in the absence of Lhβ-defensin dimer, which might be caused by the presence of the double bond in the sn2 chain of POPG. Similar results were also observed in previous studies [16, 27]. Compared with pure POPG, the order parameters of POGP decreased greatly in the presence of Lhβ-defensin dimer, especially when the POGP was around the 10 Å of defensin dimer, suggesting that large deformation of lipid structure may occur around the defensin contact regions (Fig 6). This deformation of POPG leads to water permeating inside the hydrophobic region of the bilayer. The initial contact of defensin on the surface of POPG bilayer caused expansion of membrane and hence reducing the packing density of acyl hydrocarbon chains in tail region. This resulted in water translocation in the contact region and destructed the membrane (Fig 7A). Meanwhile, the arginines of Lhβ-defensin also played important roles in the interaction between β-defensin and POPG bilayer, which leaded to strong electrostatic interactions and formed large number of hydrogen bonds with the anionic surface of POPG bilayer (Fig 7B). Thus, we speculated that the electrostatic and hydrogen bond interactions between Lhβ-defensin and membranes played important roles in the microbicidal activity of β-defensin. These results were in line with studies on human β-defensin [34, 39, 40], indicating a conserved antibacterial mechanism may exist both in fish and mammalian β-defensin.

In conclusion, a β-defensin gene was successfully cloned from soiny mullet (*Liza haematocheila*) and its expression patterns in tissues from healthy and *S*. *dysgalactiae* infected fish were investigated. The Lhβ-defensin was highly expressed in the immune-related organs of soiny mullet and up-regulated by *S*. *dysgalactiae* infection, suggesting its important role in the immune defense against bacterial. Lhβ-defensin possessed conserved monomeric structure and formed dimeric structure which was mainly stabilized by the van der Waals contacts of Leu35 and Cys37 in two anti-parallel β1-strands and the cation-π interaction between Tyr32 and Arg54 respectively in the two β1-strands. Electrostatic interactions between Arg residues and head group of POPG play important roles in the interaction of β-defensin with membrane. Hydrogen bond interactions played an important role as well. To our best of knowledge, this is the first study to analyze the structure and potential antimicrobial mechanism of fish β-defensin. Our results were useful for further studying the mechanism of antimicrobial property of fish β-defensin.

## Supporting Information

S1 Fig The cDNA and deduced amino acids of Lhβ-defensin. The signal peptide was marked by black line. The start codon (ATG) and stop codon (TAG) was boxed. The polyadenylation signal was marked by double black line.
(DOC)

S2 Fig Final snapshot of four dimmers after 20 ns MD simulation. (DOC)

S3 Fig RMSD analysis of four dimers of Lhβ-defensin. RMSD of dimer A was marked by black line, dimer B by red line, dimer C by blue line and dimer D by green line.
(DOC)

S4 Fig Buried surface of four dimers of Lhβ-defensin. Buried surface area of dimer A was marked by black line, dimer B by red line, dimer C by blue line and dimer D by green line.
(DOC)
