# Supplementary material for: Cloning and Expression of β-Defensin from Soiny Mullet (Liza haematocheila), with Insights of its Antibacterial Mechanism
Source: PLoS One. 2016 Jun 20;11(6):e0157544. doi: 10.1371/journal.pone.0157544 (PMC4913945; doi:10.1371/journal.pone.0157544)
Supplement: S1 Fig — The signal peptide was marked by black line. The start codon (ATG) and stop codon (TAG) was boxed. The polyadenylation signal was marked by double black line. (DOC) [file pone.0157544.s001.doc]

       1 tattgtatttctttctacctttatttcttttcctttttctctcatttcgcggcgcccctc
      61 ttctagtcagggccggttcctgagtcctgccgccggtcgtgcgtcgagatgatttcgtgt
     121 ctataccaatccttttcaagcagccggcgttcgtaatctgtgtttttctggccactcccc
     181 gccagaccggtctgagagcttagtggtacccaaacATGAAGGGACTGAGCTTGGTTCTCC
   1                                    M  K  G  L  S  L  V  L  L
     241 TTGTGCTTCTCCTGATTCTCGCAGCTGGAGAGGGAAATGATCCAGAAATGCAGTATTGGA

 10   V  L  L  L  I  L  A  A  G  E  G  N  D  P  E  M  Q  Y  W  T 
     301 CCTGTGGGTATAGAGGACTCTGCAGACGGTTCTGCCATGCTCAGGAATATATTGTCGGTC
 30   C  G  Y  R  G  L  C  R  R  F  C  H  A  Q  E  Y  I  V  G  H
     361 ATCATGGTTGCCCTCGGCGATACAGATGCTGTGCGGTGCGGTCTTAGcactcctccagcc
      50   H  G  C  P  R  R  Y  R  C  C  A  V  R  S  *               
     421 gtcttgtgatgcctgatcacagcatatacttgctggtaactgacaacgaatacttctgga
     481 tgcaccattttgaaatcttaaatctcacccagcccctctttggctctcaacactgagtgg
     541 gctctgataattctgttgaagtggactttatgtagttaagataaaactcacttataacta
     601 ttagatgtgttggtgcagtcatgttacccaatttgttttatgtatacattattaacaaca
     661 tcattacataatcaatcctttgtgaactctttctcctgaaatgcagtgttgccaataaat
     721 gtattaaaataaaaaaaaaaaaaaaaa

**S1 Fig. The cDNA and deduced amino acids of Lhβ-defensin.** The signal peptide was marked by black line. The start codon (ATG) and stop codon (TAG) was boxed. The polyadenylation signal was marked by double black line.
