# Supplementary figures and images for: Cloning and Expression of β-Defensin from Soiny Mullet (Liza haematocheila), with Insights of its Antibacterial Mechanism
Source: PLoS One. 2016 Jun 20;11(6):e0157544. doi: 10.1371/journal.pone.0157544 (PMC4913945; doi:10.1371/journal.pone.0157544)

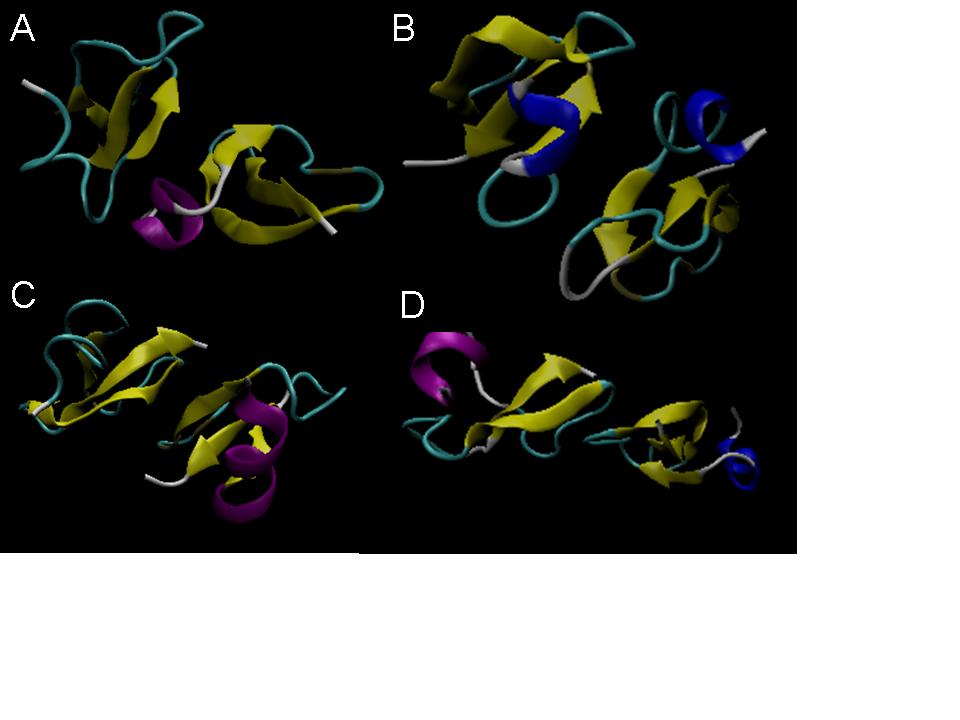


**S2 Fig. Final snapshot of four dimmers after 20 ns MD simulation**

Supplement: S2 Fig — (DOC) [file pone.0157544.s002.doc]
