# Supplementary material for: Cloning and Expression of β-Defensin from Soiny Mullet (Liza haematocheila), with Insights of its Antibacterial Mechanism
Source: PLoS One. 2016 Jun 20;11(6):e0157544. doi: 10.1371/journal.pone.0157544 (PMC4913945; doi:10.1371/journal.pone.0157544)
Supplement: S3 Fig — RMSD of dimer A was marked by black line, dimer B by red line, dimer C by blue line and dimer D by green line. (DOC) [file pone.0157544.s003.doc]

**S3 Fig. RMSD analysis of four dimers of Lhβ-defensin.** RMSD of dimer A was marked by black line, dimer B by red line, dimer C by blue line and dimer D by green line.
